# Synergistic Association between Two Alcohol Metabolism Relevant Genes and Coronary Artery Disease among Chinese Hypertensive Patients

**DOI:** 10.1371/journal.pone.0103161

**Published:** 2014-07-21

**Authors:** Yuefei Wang, Fengxia Du, Hongye Zhao, Xiaohong Yu, Jun Liu, Yu Xiao, Changzhu Lu, Xue Li, Yanli Wang, Bin Wang, Wenquan Niu

**Affiliations:** 1 Department of Physiology, Qiqihar Medical University, Qiqihar, Heilongjiang, China; 2 Department of Etiology, Qiqihar Medical University, Qiqihar, Heilongjiang, China; 3 Department of Cardiology, The First Affiliated Hospital of Dalian Medical University, Dalian, Liaoning, China; 4 The Third Division of Cardiology, Department of Internal Medicine, The Second Affiliated Hospital of Qiqihar Medical University, Qiqihar, Heilongjiang, China; 5 State Key Laboratory of Medical Genomics, Ruijin Hospital, Shanghai Jiao Tong University School of Medicine, Shanghai, China; 6 Shanghai Institute of Hypertension, Ruijin Hospital, Shanghai Jiao Tong University School of Medicine, Shanghai, China; University of Saarland Medical School, Germany

## Abstract

**Objective:**

Coronary artery disease (CAD) is a multifactorial and polygenic disease. The aim of this study was to examine the association between six polymorphisms of four alcohol metabolism relevant genes (*ADH1B*, *ADH1C*, *ALDH1b1*, *ALDH2*) and the risk of CAD in Han Chinese.

**Methods and Results:**

This was a hospital-based case-control study involving 1365 hypertensive patients. All study subjects were angiographically confirmed. Genotypes were determined with ligase detection reaction method. There was no observable deviation from the Hardy-Weinberg equilibrium for six examined polymorphisms in controls. The genotype and allele distributions of *ALDH1b1* rs2073478 and *ALDH2* rs671 polymorphisms differed significantly between the two groups (P≤0.005), even after the Bonferroni correction. The most common allele combination was A-C-C-G-C-G (alleles in order of rs1229984, rs1693482, rs2228093, rs2073478, rs886205, rs671) and its frequency was slightly higher in controls than in CAD patients (P = 0.067). After assigning the most common allele combination as a reference, allele combination A-C-C-T-C-A, which simultaneously possessed the risk alleles of rs2073478 and rs671 polymorphisms, was associated with a 1.80-fold greater risk of CAD. Further, a two-locus model including rs2073478 and rs671 that had a maximal testing accuracy of 0.598 and a cross-validation consistency of 10 (P = 0.008) was deemed as the overall best MDR model, which was further validated by classical Logistic regression model.

**Conclusion:**

Our findings provide clear evidence for both individual and interactive associations of *ALDH1b1* and *ALDH2* genes with the development of CAD in Han Chinese.

## Introduction

Coronary artery disease (CAD) is a complex disease with the interplay of multiple genetic and environmental factors precipitating its development [1]. Several risk factors for CAD have been well established including heavy alcohol consumption, family history, hypertension, diabetes and hyperlipidemia [2] and it is widely believed that CAD and its associated risk factors are largely under genetic control [3]. Given the fact that some individuals are more susceptible to CAD than others, it is of great importance to explain this inter-individual divergence in disease susceptibility in order to develop a better means for disease prediction, monitoring and personalized treatments. In this regard, Lieb and Vasan have written an overarching review on the genetic underpinnings of CAD [4]; however, there is no universal consensus on a certain gene or locus being definitively implicated in the pathogenesis of CAD. Knowing the ubiquitous nature of epistasis in determining susceptibility to common complex diseases [5], one feasible way is to examine the joint impact of multiple genes involved in a specific physiological or cellular pathway.

Alcohol metabolism exhibits considerable variation between individuals. The bulk of evidence suggests that light-to-moderate alcohol consumption plays a protective role in the development of CAD, possibly due to its potential beneficial effect on lipids, insulin sensitivity and fibrinogen profiles [6]. A major pathway for alcohol metabolism involves alcohol dehydrogenate (ADH) that catalyzes the oxidation of ethanol to acetaldehyde, and acetaldehyde dehydrogenase (ALDH) that further catalyzes the oxidation of acetaldehyde to acetate. There are three major isoenzymes of AHD, that is, ADH1a, ADH1b, ADH1c, and their encoding genes are mapped on chromosome 4q23. The human ALDH superfamily comprises 19 isoenzymes that have important physiological and toxicological functionalities. The genomic sequences of genes encoding the isoenzymes of ADH and ALDH are polymorphic and their genetic defects are observed to produce between-individual variation in the speed with which alcohol and its metabolites are degraded. Many studies have examined the association between genetic defects in ADH and ALDH isoenzymes and CAD risk; however, the results are not often reproducible [7]–[10]. As a caveat, this irreproducibility might arise from population-specific genetic heterogeneity, uncontrolled confounding and statistical underpower.

For this project, we, in a genetically homogeneous population, examined the association between six polymorphisms of four alcohol metabolism relevant genes (ADH1B, ADH1C, ALDH1b1, ALDH2) and the risk of CAD both individually and jointly among Chinese hypertensive patients.

## Methods

### Study population

This was a hospital-based case-control study involving 1365 hypertensive patients who were enrolled from the Department of Cardiology, the First Affiliated Hospital of Dalian Medical University as previously described [11], [12]. All study subjects reported no consanguinity at the time of enrollment and they were Han Chinese currently residing in Dalian, Liaoning, China. Each subject gave written informed consent before participating in our research protocol. This study received approval from the ethics committee of Dalian Medical University and was conducted in conformity with the guidelines outlined in the Declaration of Helsinki statement.

### Diagnosis

In order to avoid confounding from the existence of hypertension, only hypertensive patients were enrolled in this study. Blood pressure was measured by certified examiners with a calibrated mercury sphygmomanometer with an appropriate adult cuff size. Hypertension was defined as a systolic blood pressure over 140 mm Hg or a diastolic blood pressure over 90 mm Hg or the current usage of antihypertensive drugs. In addition, all study subjects had undergone coronary angiography, and those with the presence of over 50% stenosis in at least one of the three major coronary arteries or major branches were assigned into the CAD group. Meanwhile, hypertensive patients with normal coronary angiography (less than 50% stenosis in all arteries and branches) and a negative history of any vascular event formed the control group. Patients were excluded if they had only simple spasm of coronary arteries, myocardial bridge or other non-coronary atherosclerotic lesions.

### Sample size

According to the angiographic results, there were 679 subjects in the CAD group and 686 subjects in the control group. Subjects between the two groups were frequency-matched on age and gender.

### Measurement

At the time of enrollment, age, gender, body weight and height were recorded. For CAD patients, age referred to the first onset age of CAD. Body weight was measured after removal of shoes and wearing light clothing only. Body mass index (BMI) was calculated as weight in kilograms divided by height in meters squared.

A fasting venous blood sample was drawn from each subject. Fasting glucose was measured in fluoride plasma by an electrochemical glucose oxidase method. Lipids including triglyceride (TG), total cholesterol (TC), high-density lipoprotein cholesterol (HDL-C), low-density lipoprotein cholesterol (LDL-C) and other plasma biomarkers including blood urea nitrogen (BUN), creatinine and uric acid (UA) were measured enzymatically using available kits and auto analyzer (Sangon Biotech, Shanghai, China). Plasma high sensitivity C-reactive protein (hsCRP) level was measured using a high-sensitivity ELISA kit (Sangon Biotech, Shanghai, China).

### Genotyping

Genomic DNA was isolated from white blood cells by the phenol/chloroform extraction method and was stored at −40°C until required for batch genotyping. Plasma was prepared for quantifying routine biological profiles.

Genotypes of six examined polymorphisms were determined with the ligase detection reaction (LDR) method as previously described [13]. For each polymorphism, we synthesized two specific probes to discriminate specific bases and one common probe labeled by 6-carboxy-fluorescein at the 3′ end and phosphorylated at the 5′ end. The multiplex ligation reaction was carried out in a reaction volume of 10 µl containing 2 µl of polymerase chain reaction products, 1 µl 10×Taq DNA ligase buffer, 1 µM of each discriminating probe, 5 unit Taq DNA ligase. The ligation parameters were 30 cycles of 94°C for 30 seconds and 56°C for 3 minutes. After reaction, 1 µl of ligase detection reaction products were mixed with 1 µl ROX passive reference and 1 µl loading buffer and then were denatured at 95°C for 3 minutes and chilled rapidly in ice water. The fluorescent products of LDR were differentiated using the ABI 3730×L sequencer (Applied Biosystems, California, USA).

The accuracy of LDR method was tested in 70 randomly selected DNA samples (∼5%) which were genotyped again for quality control and were found to have complete concordance.

### Statistics

Continuous and categorical variables were compared between the two groups by the unpaired Student’s t-test and the χ^2^ test where appropriate. The Hardy-Weinberg equilibrium was tested at each locus on a contingency table of observed-versus-predicted genotype frequencies using a Fisher’s exact test. A two-tailed P value of less than 5% was considered statistically significant, whereas a value of P^b^ (0.05 divided by total number of comparisons) was considered significant after the Bonferroni correction.

For each polymorphism, we attempted three different modes of inheritance in a Logistic regression analysis, that is, additive, dominant and recessive modes after adjusting for age, gender, BMI, systolic blood pressure and fasting glucose. Statistical analyses described above were completed with the STATA software for Windows (version 11.0) (StataCorp LP, College Station, TX, USA). Study power was estimated by the Power and Sample Size Calculations (PS) software (v3.0.7).

Considering that six examined polymorphisms are located on three different chromosomes, we examined their joint effect by estimating the frequencies of allele combinations by the haplo.em program. This program computes the maximum likelihood estimates of allele combination probabilities using the progressive insertion algorithm which progressively inserts batches of loci into allele combinations of growing lengths. Only allele combination with frequency of at least 3% in all study subjects was summarized. Adjusted odds ratio (OR) and its 95% confidence interval (95% CI) for each allele combination were computed by the haplo.cc and haplo.glm programs, based on a generalized linear model [14] relative to the most common allele combination. The associated P value was simulated on 1000 replicates. Above three programs were implemented in the Haplo. Stats software (version 1.4.0) operated in the R language (version 2.14, available at the website http://www.r-project.org).

To further examine the interaction of examined polymorphisms, we resorted to an open-source multifactor dimensionality reduction (MDR) software (version 3.0.2, available at the website http://www.epistasis.org) [15], [16], a novel computational strategy for detecting and characterizing a non-linear pattern of multiple interactions in genetic association studies. All possible combinations from one- to six-locus were constructed using MDR constructive induction. A Bayes classifier in the context of 10-fold cross-validation was employed to estimate the testing accuracy of each derived best model. A single best model simultaneously had the maximal testing accuracy and cross-validation consistency (a measure of the number of times of 10 divisions of data that the best model was derived). Statistical significance was evaluated using a 1000-fold permutation test to compare the observed testing accuracies with those expected under the null hypothesis of null association. Permutation testing corrected for multiple testing by repeating the entire analyses on 1000 datasets that were consistent with the null hypothesis.

Further to test the validity of MDR method, a classical Logistic regression analysis was undertaken accordingly.

## Results

### Characteristics

Table 1 compares baseline characteristics between the CAD group and the control group. Age, gender and BMI did not differ significantly between the two groups. In contrast, the levels of systolic and diastolic blood pressures and fasting glucose were significantly higher in CAD patients than in controls (P<0.001). The percentage of patients under antihypertensive treatment was 53.02% and 30.90% in CAD patients and controls, respectively (P<0.001). CAD patients had higher levels of plasma triglyceride (P = 0.002) and hsCRP (P<0.001), but lower levels of plasma HDL-C than controls (P<0.001). No significance was attained for other biomarkers.

**Table 1:** The comparison of baseline characteristics between CAD patients and controls.

Characteristics	CAD patients(n = 679)	Controls(n = 686)	P
Age, years	64.58±8.47	64.35±9.96	0.792
Gender (males)	56.55%	54.52%	0.449
BMI, kg/m^2^	26.39±8.67	25.13±3.85	0.372
Antihypertensive treatment (%)	53.02%	30.90%	<0.001
SBP, mmHg	142.81±17.95	138.38±20.16	<0.001
DBP, mmHg	85.69±11.73	82.49±11.02	<0.001
Fasting glucose, mmol/L	6.02±2.02	5.32±1.09	<0.001
Triglyceride, mmol/L	1.98±1.11	1.77±0.96	0.002
Total cholesterol, mmol/L	4.66±1.24	4.61±0.92	0.486
HDL-C, mmol/L	1.16±0.34	1.25±0.35	<0.001
LDL-C, mmol/L	2.78±1.00	2.71±0.76	0.233
BUN, mmol/L	5.88±3.07	5.79±4.46	0.729
Creatinine, µmol/L	82.97±33.43	81.75±25.49	0.505
Uric acid, µmol/L	332.90±101.99	332.09±95.89	0.895
hsCRP, mmol/L	6.16±13.21	1.81±3.27	<0.001

*Abbreviations:* CAD, coronary artery disease; BMI, body mass index; SBP, systolic blood pressure; DBP, diastolic blood pressure; HDL-C, high-density lipoprotein cholesterol; BUN, blood urea nitrogen; hsCRP, high sensitivity C-reactive protein. Data are expressed as mean ± s.d. unless otherwise indicated.

### Individual association of six polymorphisms with CAD

Since departure from the Hardy-Weinberg equilibrium is expected in CAD patients for any polymorphism that is associated with disease status, the Hardy-Weinberg testing was only conducted in controls and no observable deviation was noted for all six polymorphisms examined.

The genotype distributions and allele frequencies of six examined polymorphisms between the CAD group and the control group are provided in Table 2. Overall, the genotype and allele distributions of *ALDH1b1* rs2073478 and *ALDH2* rs671 polymorphisms differed significantly between the two groups (P≤0.005), even after the Bonferroni correction (Significance threshold P^b^ = 0.05 divided by the total number of examined polymorphisms (n = 6): P^b^ = 0.0083). The power to reject the null hypothesis of no difference in significant allele frequencies for rs2073478 and rs671polymorphisms between patients and controls was 88.1% and 87.0%, respectively. No significance was identified for the other four polymorphisms.

**Table 2:** Genotype/allele distributions of six examined polymorphisms between patients and controls and their risk prediction for CAD.

Gene: polymorphism		CAD patients(n = 679)	Controls(n = 686)	P_χ2_	OR; 95% CI; P^*^
*ADH1B*: rs1229984	AA	329	327		0.97; 0.83–1.14; 0.755
Genotype (n):	AG	282	288	0.952	0.97; 0.78–1.20; 0.771
	GG	68	71		0.96; 0.68–1.37; 0.838
Allele (%):	G	30.78	36.07	0.752	
*ADH1C*: rs1693482	CC	650	666		1.54; 0.89–2.68; 0.126
Genotype (n):	CT	27	20	0.202	1.49; 0.83–2.56; 0.181
	TT	2	0		NA
Allele (%):	T	2.28	1.46	0.111	
*ALDH1b1*: rs2228093	CC	256	236		0.93; 0.8–1.08; 0.338
Genotype (n):	CT	302	325	0.430	0.87; 0.6901.08; 0.204
	TT	121	125		0.97; 0.74–1.28; 0.847
Allele (%):	T	40.06	41.91	0.326	
*ALDH1b1*: rs2073478	GG	239	299		1.29; 1.10–1.51; 0.001
Genotype (n):	GT	338	307	0.005	1.42; 1.14–1.77; 0.002
	TT	102	80		1.34; 0.98–1.83; 0.068
Allele (%):	T	39.91	34.04	0.001	
*ALDH2*: rs886205	CC	472	488		1.03; 0.83–1.28; 0.778
Genotype (n):	CT	199	184	0.293	1.08; 0.86–1.36; 0.512
	TT	8	14		0.57; 0.24–1.37; 0.211
Allele (%):	T	15.83	15.45	0.785	
*ALDH2*: rs671	GG	324	367		1.27; 1.08–1.48; 0.003
Genotype (n):	GA	259	258	0.005	1.26; 1.02–1.56; 0.033
	AA	96	61		1.69; 1.20–2.37; 0.003
Allele (%):	A	33.21	27.70	0.002	

*Abbreviations:* CAD, coronary artery disease; OR, odds ratio; 95% CI, 95% confidence interval. *The upper risk estimates were calculated under an additive mode (major homozygotes versus heterozygotes versus minor homozygotes), the middle under a dominant mode (major homozygotes versus heterozygotes plus minor homozygotes) and the lower under a recessive mode (major homozygotes plus heterozygotes versus minor homozygotes).

The risk prediction of six examined polymorphisms for CAD was examined under three genetic modes of inheritance (Table 2). Likewise for *ALDH1b1* rs2073478, the odds of having CAD was increased by 29% (95% CI: 1.10–1.51; P = 0.001) under an additive mode and 42% (95% CI: 1.14–1.77; P = 0.002) under a dominant mode, even with a Bonferroni corrected alpha of 0.05/6. With regard to *ALDH2* rs671, the odds of CAD was only significant under additive (OR = 1.27; 95% CI: 1.08–1.48; P = 0.003) and recessive (OR = 1.69; 95% CI: 1.20–2.37; P = 0.003) modes after the Bonferroni correction.

### Joint association of six polymorphisms with CAD: allele combination

Table 3 compares the frequencies of derived allele combinations from six polymorphisms between the CAD group and the control group. The most common allele combination was A-C-C-G-C-G (alleles in order of rs1229984, rs1693482, rs2228093, rs2073478, rs886205, rs671) and its frequency was slightly higher in controls than in CAD patients (27.36% versus 25.57%, Simulated P = 0.067). Another allele combination A-C-C-T-C-A was overrepresented in CAD patients relative to controls and differed significantly between the two groups (Sim P<0.001) even with a Bonferroni corrected alpha of 0.05/11 and the estimated power to reject the null hypothesis of no difference between the two groups was 99.1%.

**Table 3:** Estimated frequencies of common allele combinations (≥3%) of six examined polymorphisms and their risk prediction for CAD.

Allele combination	All subjects	Patients	Controls	Sim P	OR; 95% CI; P
A-C-C-G-C-G	26.54	25.57	27.36	0.067	Reference
A-C-C-T-C-G	13.62	12.55	14.78	0.520	0.94; 0.67–1.33; 0.735
A-C-C-G-C-A	9.48	10.06	9.38	0.264	1.29; 0.85–1.94; 0.228
A-C-C-T-C-A	7.33	9.32	5.00	<0.001	1.80; 1.19–2.71; 0.005
G-C-T-G-T-G	6.76	6.28	7.13	0.286	0.98; 0.63–1.52; 0.936
G-C-T-G-C-G	6.06	5.56	6.54	0.245	0.92; 0.6–1.41; 0.701
A-C-T-G-C-G	4.41	4.03	5.02	0.019	0.85; 0.51–1.42; 0.544
G-C-T-G-C-A	3.74	3.33	4.44	0.183	0.79; 0.44–1.41; 0.427
G-C-T-T-T-G	3.71	3.93	3.48	0.491	1.92; 1.11–3.30; 0.019
G-C-T-T-C-G	3.64	4.68	2.73	0.085	1.25; 0.71–2.19; 0.433
G-C-T-G-T-A	3.13	2.67	3.18	0.636	0.73; 0.37–1.44; 0.363

*Abbreviations*: CAD, coronary artery disease; Sim P, simulated P value; OR, odds ratio; 95% CI, 95% confidence interval.

After assigning the most common allele combination as a reference, allele combination A-C-C-T-C-A, which simultaneously possessed the risk alleles of rs2073478 and rs671 polymorphisms, was associated with a 1.80-fold greater risk of CAD, whereas this association was nonsignificant after the Bonferroni correction (P^b^ = 0.05/11). There was no observable significance for the other allele combinations between the two groups.

Taking all allele combinations as a whole, there was a significant association with plasma HDL-C (Simulated P<0.001) in CAD patients and plasma TC (Simulated P = 0.031) in controls (Table 4).

**Table 4:** Global testing of all derived allele combinations with anthropometric indexes and clinical biomarkers in both CAD patients and controls.

Characteristics	CAD patients		Controls	
	Score	Sim P	Score	Sim P
Age, years	27.44	0.334	21.05	0.456
Gender	30.56	0.167	13.01	0.908
BMI, kg/m^2^	19.11	0.746	19.47	0.236
SBP, mmHg	22.91	0.526	26.74	0.221
DBP, mmHg	17.71	0.817	19.04	0.643
Fasting glucose, mmol/L	18.64	0.814	20.45	0.487
Triglyceride, mmol/L	10.49	0.981	9.73	0.982
Total cholesterol, mmol/L	10.49	0.981	34.57	0.031
HDL-C, mmol/L	54.89	<0.001	13.37	0.895
LDL-C, mmol/L	27.94	0.218	31.90	0.072
BUN, mmol/L	24.47	0.435	28.65	0.103
Creatinine, µmol/L	9.78	0.995	9.03	0.993
Uric acid, µmol/L	27.77	0.527	12.11	0.937
hsCRP, mmol/L	5.08	0.998	10.38	0.887

*Abbreviations*: CAD, coronary artery disease; Sim P, simulated P.

### Joint association of six polymorphisms with CAD: interaction analysis

Interaction analysis of six examined polymorphism by MDR software is summarized in Table 5. A two-locus model including rs2073478 and rs671 polymorphisms that had a maximal testing accuracy of 0.598 and a cross-validation consistency of 10 out of 10 was deemed as the overall best MDR model, which was significant at a level of 0.008, indicating that a model this good or better was observed only by 8 out of 1000 permutations and was thus unlikely under the null hypothesis of null association.

**Table 5:** Summary of MDR analysis.

Best combination of each model	CV consistency	Testing accuracy	P
rs2073478	9	0.578	0.016
rs2073478, rs671	10	0.598	0.008^*^
rs2073478, rs671, rs1229984	7	0.569	0.052
rs2073478, rs671, rs1229984, rs886205	9	0.567	0.191
rs2073478, rs671, rs1229984, rs886205, rs2228093	8	0.539	0.288
rs2073478, rs671, rs1229984, rs886205, rs2228093, rs193482	10	0.543	0.267

*Abbreviations*: MDR, multifactor dimensionality reduction; CV, cross validation. *Overall best MDR model.

To test the validity of MDR method, we further conducted a classical Logistic analysis that incorporated the produce of two polymorphisms in the best model and confounding factors including age, gender, BMI, systolic blood pressure and fasting glucose. The interaction of these two polymorphisms was found to be associated with a 1.16-fold (95% CI: 1.09–1.24; P<0.001) increased risk of CAD under an additive mode.

## Discussion

The aim of this study was to examine the association between six polymorphisms of four alcohol metabolism relevant genes and CAD risk in northeastern Han Chinese. It is worth noting that genetic defects of *ALDH1b1* and *ALDH2* might not only play a leading role in the prediction of CAD susceptibility, but also interact synergistically to enhance the power of risk prediction. Our findings provide convincing evidence for the potential involvement of alcohol metabolism relevant genes in the pathogenesis of CAD.

Three recent meta-analyses consistently supported a contributory role of *ALDH2* rs671 polymorphism in susceptibility to CAD and myocardial infarction, with the rs671-A allele associating with an increased risk of at least 1.2 and 1.32 [17]–[19]. Moreover, another study by Guo et al in Han Chinese found that the odds of CAD for carriers of rs671 A allele reached as high as 1.85 in the entire study cohort and 1.95 in non-drinkers, which was reasoned to be due to the changes of plasma HDL-C and endothelial asymmetric dimethylarginine levels [20]. This significant observation was in agreement with the results of our single-locus analysis. Additionally, we observed that this polymorphism might be inherited in a recessive mode, as the effect estimates were comparable between additive (OR = 1.27) and dominant (OR = 1.26) modes, and subjects homogeneous for the rs671-A allele were 69% more likely to develop CAD compared with those with the rs671-G allele. The genetic distributions of rs671 exhibited strong ethnic heterogeneity with its mutant allele common in East Asians (30–50%) yet rare in Caucasians (<5%). This heterogeneity reflects either diverse genetic backgrounds or different linkage disequilibrium patterns across different ethnical groups. It is generally believed that a polymorphism may be in close linkage with another nearly causal locus in one ethnic group but not in another. In view of the existence of ethnic heterogeneity, there is an urgent need to construct a database of CAD-susceptibility genes or polymorphisms in each ethnic group.

Besides the validation of significant susceptibility of *ALDH2* rs671 polymorphism to CAD, we additionally identified a synergistic interaction between rs671 and another individually significant polymorphism rs2073478 in *ALDH1b1*. On one hand, this interaction was reflected in our allele combination analysis, as the combination simultaneously possessing the two ‘risk’ alleles of these two polymorphisms was associated with a greater risk of CAD, which was further observed to be mediated via regulating lipid profiles by our allele combination-phenotype analysis. In fact, previous studies have shown that the mechanisms of alcohol involved in CAD might be explained by an effect on increasing plasma HDL-C level and decreasing platelet aggregation [21], [22], as well as a direct effect on ischaemic myocardial or through an anti-inflammatory mechanism [23], [24]. It is thereby reasonable to speculate that if involved, rs671 and rs2073478 polymorphisms might be interactively implicated in the pathogenesis of CAD by regulating lipid profiles. On the other hand, the interaction between *ALDH2* rs671 and *ALDH1b1* rs2073478 polymorphisms was identified as the overall best synergistic model by using a promising data-mining analytical method, MDR, which reinforced the results of both individual and allele combination analyses. This method is nonparametric and genetic model-free nature in design [25] and has been successfully applied to detect high-order gene-gene and gene-environment interactions in case-control and discordant-sib-pair studies with relatively small samples [26], [27]. In fact, we have recently employed the MDR method to characterize potential synergism between multiple polymorphisms of advanced glycation end-product receptor gene [12] and DNA repair relevant genes [11] in association with CAD in the present study population. Such interaction cannot be overlooked in genetic association studies because no one gene or its polymorphism is functional in an isolated environment and it is critical to identify those genes that interact with one another and to determine how this interaction occurs. So genotyping data from alcohol mechanism relevant genes incorporating the joint and synergistic analytical strategies would facilitate the identification of subjects at high risk of developing CAD in future clinical screening.

Several specific strengths and limitations of this study merit adequate consideration. A major advantage was that both CAD patients and controls were angiographically confirmed, which can minimize the misclassification of study subjects as far as possible. A further merit was the selection of hypertensive patients as a background population, which can remove the confounding impact of hypertension, considering that hypertension is an independent predisposing factor for CAD [28]. However, this study was limited by the cross-sectional design, which did not allow us to assess the time-course in the association between alcohol metabolism relevant genes and CAD. The second limitation was that data on alcohol consumption were not available, which prevented further stratified analyses by this index. The third limitation was that only six polymorphisms of four alcohol metabolism relevant genes were analyzed, and it is of added interest to explore other candidate genes in the metabolism of endogenous active aldehydes such as 4-hydroxynonenal. The fourth limitation was that all study subjects involved were older than 50 years and further large association studies in a young population of CAD patients are of specific interest, as genetic factors may exhibit greater contribution to those suffering premature CAD and in the absence of strong environmental risk factors [29]. The fifth limitation was despite robust in characterizing synergism, MDR method has some inherent drawbacks, such as computational intensiveness, indistinct interpretation, lack of sensitivity and heterogeneity-free assumption [25], [30]. The sixth limitation was the fact that our study subjects were of Han Chinese descent limited the generalizability of our findings, calling for further confirmation in other ethnic groups.

To conclude, our findings provide clear evidence for the individual and interactive associations between genetic defects of *ALDH1b1* and *ALDH2* and the development of CAD in northeastern Han Chinese. For practical reasons, large, well-designed longitudinal studies attempting to shed light on gene-to-gene and gene-to-environment interactions, as well as studies striving to provide biological or clinical implications of alcohol metabolism relevant genes, are needed in future investigations.
